# One-Step Preparation of Carboxymethyl Cellulose—Phytic Acid Hydrogels with Potential for Biomedical Applications

**DOI:** 10.3390/gels8100647

**Published:** 2022-10-12

**Authors:** Alina Ghilan, Loredana Elena Nita, Daniela Pamfil, Natalia Simionescu, Nita Tudorachi, Daniela Rusu, Alina Gabriela Rusu, Maria Bercea, Irina Rosca, Diana Elena Ciolacu, Aurica P. Chiriac

**Affiliations:** 1 Department of Natural Polymers, Bioactive and Biocompatible Materials, “Petru Poni” Institute of Macromolecular Chemistry, 700487 Iasi, Romania; 2 Department of Polymer Physical Chemistry, “Petru Poni” Institute of Macromolecular Chemistry, 700487 Iasi, Romania; 3 Centre of Advanced Research in Bionanoconjugates and Biopolymers, “Petru Poni” Institute of Macromolecular Chemistry, 700487 Iasi, Romania; 4 Department of Physics of Polymers and Polymeric Materials, “Petru Poni” Institute of Macromolecular Chemistry, 700487 Iasi, Romania

**Keywords:** carboxymethyl cellulose, phytic acid, hydrogels, biomedical applications

## Abstract

Hydrogels based on natural, biodegradable materials have gained considerable interest in the medical field due to their improved drug delivery profiles and tissue-mimicking architecture. In this regard, this study was devoted to the preparation and characterization of new physically crosslinked hydrogels based on carboxymethyl cellulose and an unconventional crosslinking agent, phytic acid. Phytic acid, in addition to its antioxidant and antibacterial effects, can improve the biological properties and stability of gels, without adding toxicity. Fourier transform infrared (FTIR) spectroscopy, rheological studies and thermal analysis confirmed the hydrogel formation. The influence of the ratio between the cellulose derivative and the crosslinker upon the morphological structure and water uptake was evidenced by scanning electron microscopy (SEM) and swelling measurements in simulated body fluids. Furthermore, procaine was entrapped within the hydrogels and used as a model drug for in vitro studies, which highlighted the dependence of the drug release on the phytic acid content of the matrix. The materials demonstrated antibacterial effects against *Escherichia coli* and *Staphylococcus aureus* bacteria. The biocompatibility was assessed on fibroblast cells, and according to our results, hydrogels can improve cell viability highlighting the potential of these systems as therapeutic scaffolds for skin tissue engineering.

## 1. Introduction

Carboxymethyl cellulose (CMC), one of the most important polysaccharides, is an anionic, water-soluble derivative of cellulose [1]. It has attracted considerable scientific attention and is widely used in a variety of biomedical applications due to its inherent properties such as biocompatibility, biodegradability, tissue resemblance, enhanced water solubility, cost-effectiveness, high stability, non-toxicity and wide range of possible chemical reactions [2]. Among the most attractive biomedical applications, we list: drug delivery [3,4], tissue engineering [5], wound dressing [6], diagnosis of various diseases [7], bone tissue engineering [8], sensing matrices [9], dental materials [10], ophthalmic applications [11] and bioinks for 3D printing [12,13]. These applications revealed by the literature emphasize the appealing possibility of extending CMC’s applicability to new and unconventional uses. Moreover, pristine CMC has been shown to be compatible with mucous membranes, bone and skin and can maintain a moist environment in the areas around a wound, which will help increase the extracellular matrix (ECM) and reepithelialization [2]. Additionally, it can activate macrophages and increase cytokine levels in wounds [14]. In this regard, this study presents a new approach for using CMC as a starting material in the preparation of a novel hydrogel that could be applied as a dressing material.

Hydrogels are three-dimensional polymer networks that can hold large amounts of water, have flexible physical properties and have the ability to be processed into various shapes and sizes. They are also similar to natural tissues and can protect unstable drugs from degradation [15]. However, although numerous types of hydrogels have been developed, the extending of their applications area has some limitations resulting from the complex, irreproducible processing techniques, as well as the synthesis of hydrogels using toxic materials [16]. It is important to add that in the case of CMC, epichlorohydrin is often used, and studies have shown that it can produce a large amount of poisonous and carcinogenic byproducts in strong alkaline conditions [17]. 

Therefore, this study focused on obtaining a nontoxic, eco-friendly, highly biodegradable hydrogel from an edible crosslinker—phytic acid (PA)—by using a simple processing method that does not require complicated procedures or synthetic reagents other than CMC, PA and water. 

PA, also known as inositol hexakisphosphate or inositol polyphosphate, is a major component of plant seeds and can be considered a promising candidate for naturally crosslinking scaffolds. This compound has many hydroxyl-bearing phosphoric groups and exhibits excellent antioxidant and chelating capabilities. Crosslinking between PA and natural polymers could happen upon bonding anions of PA to cations of such a polymer, but also through the formation of hydrogen bonds due to the presence of hydroxyl groups within the structure of the compound [18]. Thus, PA presents versatility in obtaining of functional materials for medical applications. In the literature, this molecule has been crosslinked with polymers such as alginate [19], chitosan [20], carboxymethyl chitosan [21], poly(vinyl alcohol) [22], poly((trimethylamino)ethyl methacrylate chloride) [23], polyacrylamide/chitosan [24] and polyaniline [25] to create various hydrogels for biomedicine. To avoid the conventional application of crosslinkers with higher cytotoxicity, the authors agreed on the significance of using naturally derived crosslinking agents. Moreover, PA has a wide range of biological activities, such as antibacterial, antidiabetic, anti-inflammatory, anticancer, antioxidant, antiangiogenic, antiulcer, antiviral, hypoallergenic, lipid-lowering, immunomodulatory and neuroprotective effects [26]. 

Due to its countless proven properties, an intensive investigation of the synthesis and characterization of CMC/PA hydrogels is being carried out in the present paper. Additionally, in order to exploit their properties that recommend them as potential drug delivery systems, procaine was chosen as a hydrophilic model drug. Procaine (P) is a local anesthetic known to reduce bleeding and accelerate the wound-healing process, and it has the ability to treat a large variety of infections [27].

## 2. Results and Discussions

**Figure 1:**
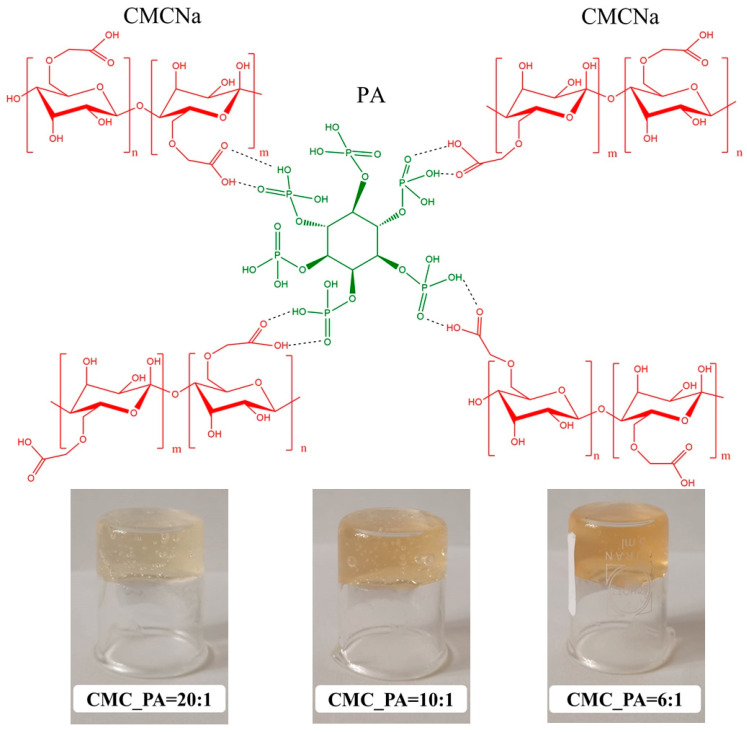
Schematic illustration for the formation of CMC/PA hydrogels and the inverted vial test.

The addition of PA to CMC promoted the formation of hydrogels stabilized by intermolecular bonds between the functional groups of both compounds, which are schematically exemplified in Figure 1. Moreover, as can be seen from the vial inversion test, stable gels are formed at all proposed ratios between the polysaccharide and PA. 

### 2.1. FTIR Spectroscopy Analysis

**Figure 2:**
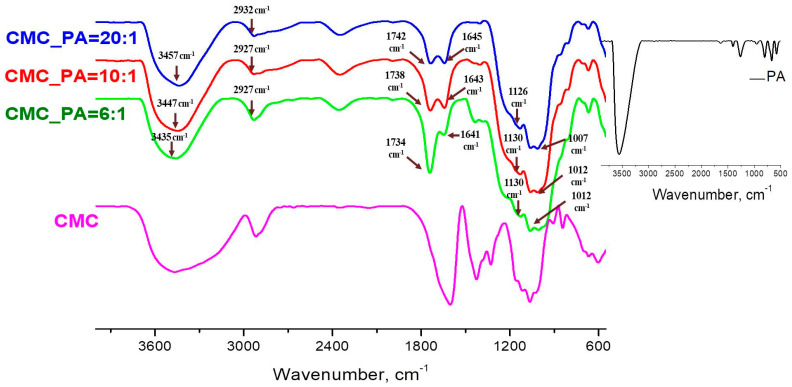
FTIR spectra of the hydrogels of CMC/PA and pure CMC; the spectrum of PA can be found in the insert.

Figure 2 shows the FTIR analysis of the CMC and CMC/PA hydrogels, while the PA spectrum is found in the inset. The FTIR spectrum of pure CMC shows a broad absorption band at 3464 cm^−1^, due to the stretching frequency of the –OH groups, while the stretching vibration of the C−H groups can be seen at 2916 cm^−1^. A strong absorption band related to carboxylates’ COO− asymmetric (1605 cm^−1^) and symmetric stretches (1425 cm^−1^) and a band at 1329 cm^−1^ attributed to CH_2_ scissoring were recorded. An absorption band with multiple peaks in the 1113–1004 cm^−1^ range is attributed to the ether bonds in the cellulose backbone [28]. The most representative bands in the PA spectrum can be found at 1632 cm^−1^ and 1401 cm^−1^, attributed to the stretching frequencies of the P=O groups, and at 1265 cm^−1^, attributed to the frequencies of the P–O–C groups. The distant band at 3650–3200 cm^−1^ is attributed to –OH vibrations [29]. 

The IR spectra of the CMC/PA structures clearly show the presence of the characteristic bands of their parent components, and thus both compounds participate in the formation of the gel (Table 1). As a general trend for the characterized hydrogels, it can be observed that the broad band in the region 3650–3200 cm^−1^ attributed to the OH vibration shifts to lower wavelengths with the increase of the PA content in the mixture. Moreover, the stretching frequencies of the P=O groups, as well as the anti-symmetric and symmetric frequencies of the P–O–C groups, move also to lower frequencies. These shifts demonstrate the occurrence of intermolecular bonds between the CMC and the crosslinking agent. The OH band of the associated species absorbs at lower wavenumbers because of the fact that the formation of the hydrogen bonds weaken the OH band, thus indicating an increase in the interactions between CMC and PA. Therefore, a high feed ratio of PA determines a greater crosslinking of CMC by PA [30]. Another area of interest that shows changes, an increase in the intensity of the peaks and a shift towards higher wavelengths, is represented by the characteristic region of the COO− groups in CMC. Thus, in the spectra of hydrogels, the carboxylate band disappears (1603 cm^−1^) and is replaced by a band at 1742 cm^−1^ attributed to the carboxylic acid group COOH. This behavior can be explained by the substitution of Na^+^ by H^+^ in the CMC polymer chains during the acidification promoted by PA, which acts as well as an ion donor. We can conclude that the addition of PA to CMC engendered the formation of hydrogels stabilized by intermolecular bonds between the functional groups of both compounds.

### 2.2. Swelling Tests

**Figure 3:**
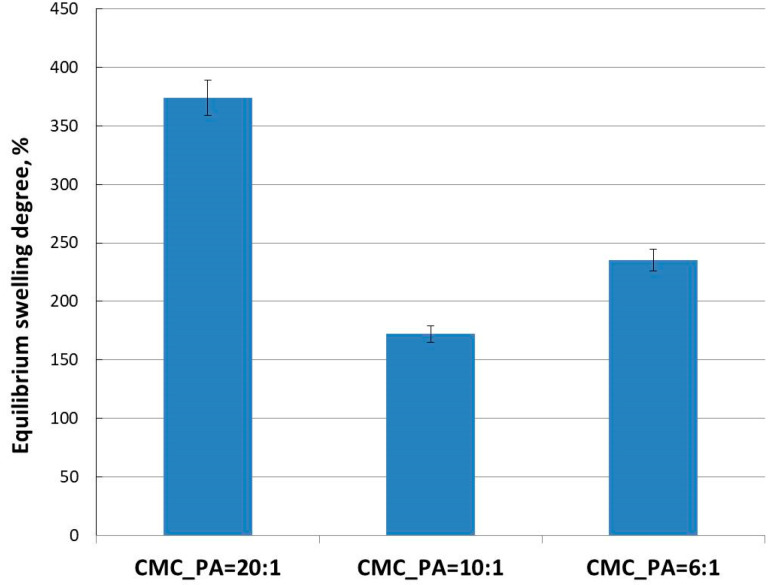
Equilibrium degree of swelling of the hydrogels at different CMC/PA molar ratios.

Figure 3 illustrates the equilibrium degree of swelling (SDE) of the CMC/PA hydrogel samples with various amounts of PA crosslinker. The compounds exhibited high SDE, as they absorbed the phosphate buffer solution at a pH = 7.4. By comparing the degree of swelling of the samples, a lower absorption is observed as the amount of crosslinker increases. However, although the highest amount of buffer is absorbed at the lowest PA content (CMC_PA = 20:1, SDE = 374%), the sample with the lowest SDE was CMC_PA = 10:1 (SDE = 172%) and not CMC_PA = 6:1 (SDE = 235%), as was expected. Thus, it follows that the optimal ratio at which a stable network is obtained is 10:1. An increase in the amount of the crosslinker results in a network with larger and more irregular meshes, a statement that will be demonstrated by SEM microscopy. 

Taken together, these results demonstrated that the hydrophilicity of CMC/PA hydrogels can vary in correlation with the CMC/PA ratio. Considering that swelling behavior is crucial for promoting a moist microenvironment that aids the wound-healing process, they may be therefore suitable for wound dressings and skin tissue substitutes.

### 2.3. Rheological Studies

**Figure 4:**
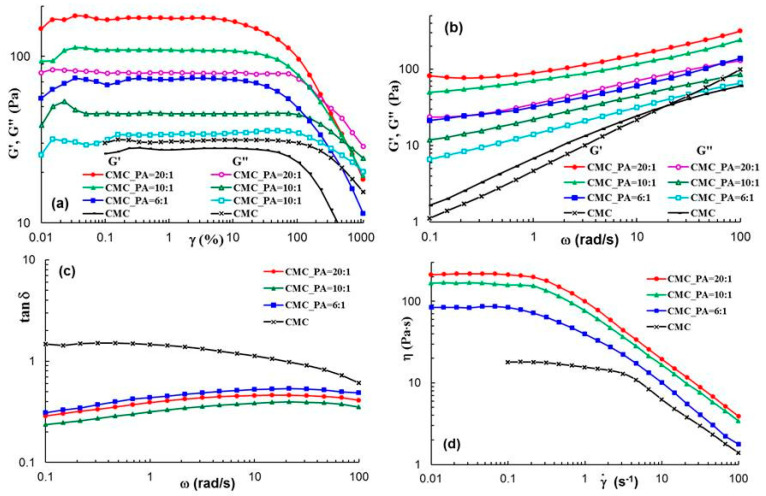
Rheological behavior of the samples in different shear conditions: amplitude sweep (**a**), frequency sweep (**b**,**c**) and shear flow (**d**) at 25 °C.

Figure 4 presents the rheological behavior of the gels in different shear conditions. In the linear range of viscoelasticity, the elastic (G′) and viscous (G″) moduli are independent of the strain (γ) (Figure 4a). Gel-like structure is depicted for all CMC/PA samples: G′ > G″ (Figure 4a,b), and tanδ < 1 (Figure 4c); in similar conditions, CMC behaves as a structured fluid (G′~ω^0.66^ and G″~ω^0.57^). G′ and G″ present the highest values for the sample CMC_PA = 20:1. Additionally, the values of the shear viscosity are higher as CMC content increases (Figure 4d). The sample morphology (the crosslinking density and the pore distribution) influences the rheological behavior. All CMC/PA samples present tanδ < 1, whereas for CMC, tanδ > 1 at low and moderate values of oscillation frequency. However, CMC_PA = 10:1 shows the highest degree of viscoelasticity, i.e., the maximum difference between the elastic and viscous moduli (minimum values of tanδ, Figure 4c).

The flow curves shown in Figure 4d show a Newtonian region at low shear rates and a non-Newtonian behavior for moderate and high shear rates when the shear viscosity scales as ${\dot{\gamma }}^{-(0.66÷0.7)}$.

### 2.4. Scanning Electron Microscopy Analysis

**Figure 5:**
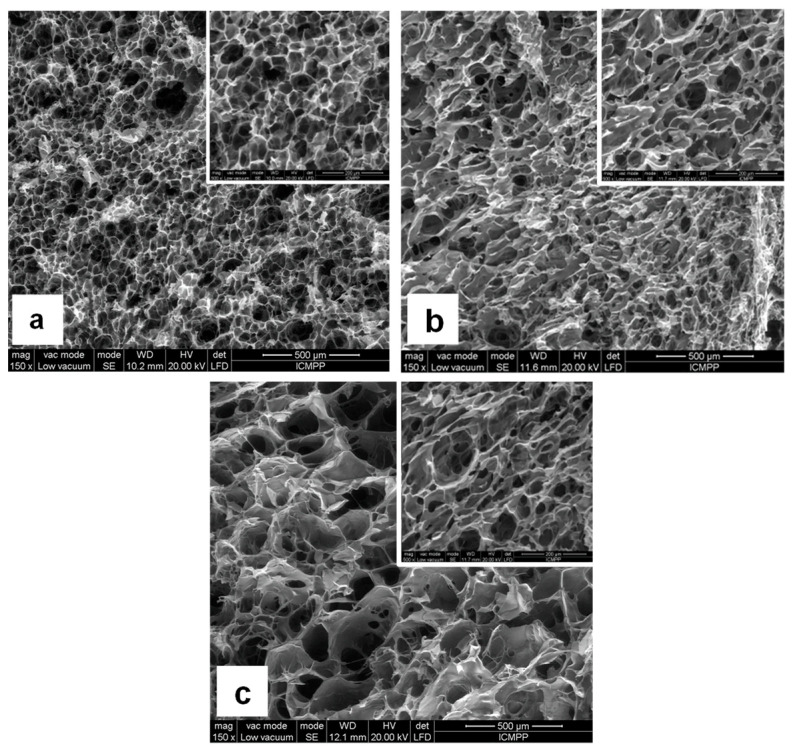
SEM micrographs of freeze-dried hydrogels: (**a**) CMC_PA = 20:1; (**b**) CMC_PA = 10:1; (**c**) CMC_PA = 6:1.

In order to decode the relationship between the material structure and the capacity for loading and subsequent release of drugs from hydrogels with different crosslinker content, SEM images of freeze-dried gels were obtained (Figure 5). A highly porous network with fine and interconnected pores was observed for all three samples, allowing the absorption of a large amount of solvent. This interconnected pore structure facilitates the diffusion of the solvent molecules in and out through it. This aspect is also in line with the requirements for soft tissue engineering, where it is desirable for the pores to be highly interconnected to aid cell proliferation, differentiation and subsequent tissue formation [31].

Furthermore, an increase in the crosslinking density induces changes in the morphology of the network. Therefore, in the case of the sample with the highest amount of crosslinker (CMC_PA = 6:1), a distinct morphology could be observed compared to the other two samples, with the network showing larger and more irregular pores.

### 2.5. Thermal Behavior

**Figure 6:**
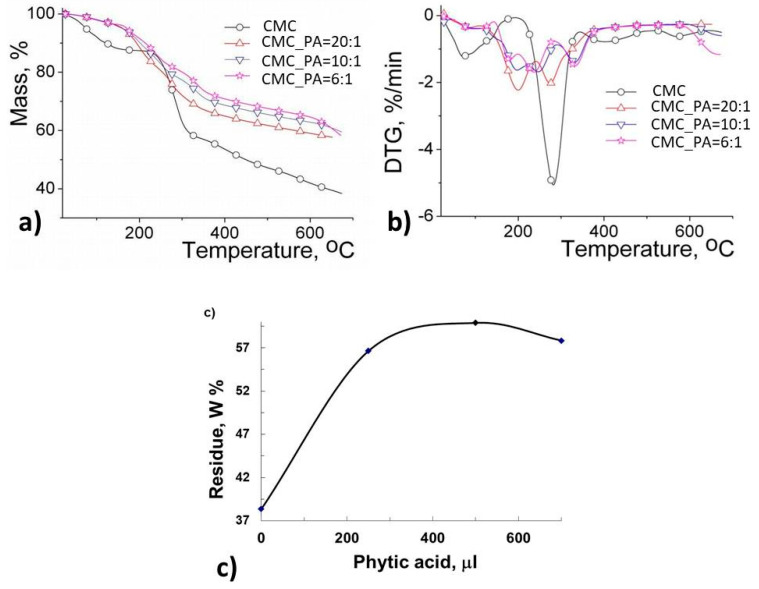
(**a**) TG and (**b**) DTG curves of CMC and CMC/PA hydrogels and (**c**) the residual percentage depending on the amount of PA in the synthesis.

Figure 6 exhibits primary thermograms (TG) and derivative thermograms (DTG) for CMC and CMC-based hydrogels, while the main thermal parameters are included in Table 2. The thermal degradation of CMC and CMC hydrogels takes place in four stages. At the beginning of the decomposition process (first stage), mass losses are around 12.55% for CMC and from 2.4–4.55% in the case of the hydrogels, these losses being a consequence of moisture removal and evaporation of bound water [32]. This process takes place up to a temperature of about 230 °C for CMC and 168 °C for the gels. In the second stage of the decomposition, the reduced mass loss in hydrogels is due to the physical crosslinking bridges occurring between CMC and PA, while for CMC it is related to the depolymerization of glycosyl units to volatile species by the cleavage of the chains [33]. This process is favored by the reactions of intramolecular and intermolecular rearrangements, resulting in a biochar formation with highly arranged structure and thermal stability. The weight loss was 29.96% for CMC, while for gels it varied between 4.48 and 15.93%, depending on the PA ratio in the hydrogel systems. The mass losses occurring in the third stage of degradation are attributed to pyrolytic decomposition by breaking the C–O–C bonds present in the glycosidic ring of CMC, degradation of the inositol structure in PA and depolymerization processes and degradation of the side groups [34]. In the last stage of degradation (the fourth stage), the main weight losses are attributed to the fragmentation of molecules associated with pyrolytic decomposition leading to the formation of aromatic units and the decomposition of carbonaceous residues. From the above presented data, we can conclude that the CMC/PA hydrogels are more thermally stable than the native CMC (as seen from the T_20_ and T_25_ values in Table 2). By representing the amount of residue left after thermal decomposition according to the PA content (Figure 6c), a better thermal stability is observed in the case of the CMC_PA = 10:1 sample, thus resulting in the fact that the gels present an increased number of physical interactions that induce higher stability in the polymer network, behavior in good agreement with the swelling data (Figure 3).

### 2.6. In Vitro Studies of Procaine Release

**Figure 7:**
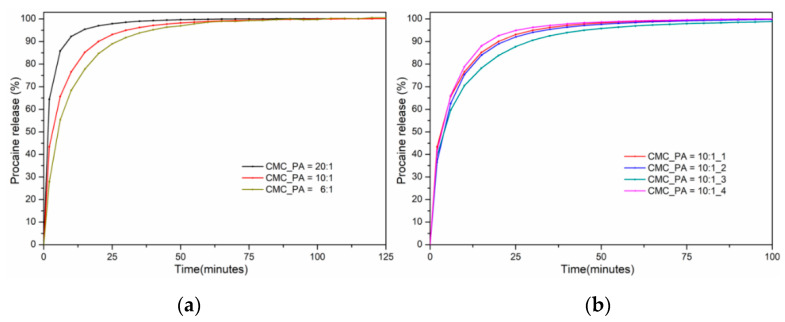
Procaine release profiles from the investigated hydrogels with different compositions (**a**) and different drug concentrations (**b**).

The in vitro release profiles of procaine from the CMC/PA hydrogel samples under simulated physiological conditions (T = 37 °C, pH = 7.4) are shown in Figure 7. A typical biphasic release pattern is observed, namely a burst release followed by a slower sustained release. This can be explained by the highly hygroscopic nature of CMC as a result of its hydrophilic carboxylate groups in the polymer backbone, which causes a rapid hydration of the network, followed by a fast release of the drug. As a general finding, the drug release results showed a similar trend to that of the swelling results. 

The fastest release of procaine, with an equilibrium reached after 65 min, was recorded in the case of the CMC_PA = 20:1 hydrogel. This may be due to the ionic contribution of CMC in the buffer solution at pH 7.4, as well as to the use of a smaller amount of crosslinking agent in the synthesis of this material, which led to the establishing of a low number of crosslinking bonds and implicitly to the obtaining of a more relaxed structure. The drug release equilibrium of the bioactive substance from both hydrogels CMC_PA = 10:1 and CMC_PA = 6:1 was reached after 90–190 min. In the case of CMC_PA = 10:1, this is attributed to the presence of a larger amount of crosslinking agent in the preparation process that induces an increase in the crosslinking density and the formation of a polymer network with smaller meshes, which maintain the incorporated bioactive compound more firmly through physical bonds. As a consequence, the swelling of the hydrogels decreases, which leads to a delay in the release of the drug. 

Figure 7b shows the samples with an optimal ratio of CMC/PA = 10:1 and different amounts of incorporated drug. It can be noted that the sample with the smallest amount of drug, CMC_PA = 10:1_4, releases the fastest. Thus, it can be concluded that with an increase in the drug content, additional intra- and intermolecular interactions between the matrix and the bioactive component appear. The slowest release (k = 0.278 min^−n^), with a behavior closer to a Fickian release, is shown by the sample CMC_PA = 10:1_3. It can be assumed that at this ratio there is an optimal balance of physical interactions that determines a delayed release.

The value of the release exponent (n) is less than 0.5 (Table 3) for CMC_PA = 20:1 and CMC_PA = 10:1, suggesting a “pseudo-Fickian” diffusion mechanism, caused by a higher rate of polymer relaxation in comparison with the drug diffusion rate. The CMC_PA = 6:1 sample recorded an “n” value of 0.568, which is appropriate for a non-Fickian release behavior. Additionally, it was found that raising the crosslinking agent’s concentration causes an increase in the “n” value. The release rate constant (k) values decreased with the increase of the PA content used for the sample preparation. The sample CMC_PA = 20:1 registered the highest value, k = 0.553 min^−n^, which is correlated to the most accelerated rate of procaine release in comparison with the other samples.

### 2.7. In Vitro Biocompatibility Assay 

**Figure 8:**
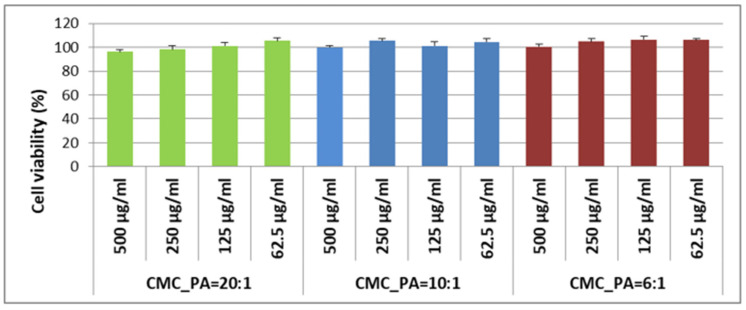
Cell viability of normal human dermal fibroblasts exposed to hydrogel extracts (500/250/125/62, 5 µg/mL) for 24 h; experiments were done in triplicate, and treated cell viability was expressed as percentage of control cells’ viability; graphical data were expressed as means ± standard error of the mean.

The biocompatibility of the hydrogels was evaluated in fibroblast cells using an MTS assay to further validate their potential to be applied as skin repair materials. The in vitro experiments were carried out in accordance with the ISO10993-5 standard test method (indirect contact) [35]. Figure 8 illustrates the cell viability after incubation with extracts from CMC/PA hydrogels at different concentrations. The MTS results corresponding to the extracts of all analyzed samples revealed that the cell viability of the fibroblast cells was higher than 96% at all concentrations of the extraction media after 24 h, similar to the reference control condition (100%, within statistical variation). Even though the percentage is very small, the data show that adding a larger amount of PA improves cell viability to some extent, demonstrating once again the advantages of this natural crosslinker. To conclude, all samples demonstrated equivalent cytocompatibility toward fibroblast cells, and this fact can be attributed to the high biocompatibility of CMC and PA. Thereby, these hydrogels have properties and in vitro cytocompatibility suitable for prospective applications in wound healing.

### 2.8. Antimicrobial Activity

The antimicrobial activity was evaluated against two different microorganisms using the Kirby–Bauer test, also known as the disk-diffusion method. The diameters of the inhibition zones are presented in Table 4. The hydrogels were effective against both the Gram-positive bacterial strain *S. aureus* and the Gram-negative bacterial strain *E. coli*. These data are in agreement with previous results from the literature reporting that PA exhibits significant inhibitory effects on bacteria and biofilm development when combined with other compounds [36,37]. Additionally, the diameter of the inhibition zone increased slightly with increased amounts of PA in the hydrogel. These results highlighted that PA positively influences the properties of these materials, which could be exploited in biomedical applications where novel strategies to counter antimicrobial resistance are required.

## 3. Conclusions

This study focused on the preparation of new hydrogels based on carboxymethyl cellulose and phytic acid through a facile and cost-effective method, as well as their comprehensive characterization for subsequent use as possible skin repair substitutes. The influence of the ratio between the cellulose derivative and the natural and biocompatible crosslinking agent, phytic acid, on the properties of the new compounds was highlighted. The formation of intermolecular bonds between the two compounds was evidenced in the FTIR spectra by the shifts of the absorption bands characteristic to the hydroxyl groups to lower wavenumbers. Furthermore, these interactions proved to be stronger with the increase of the content of crosslinker agent content in the mixture. These results were also supported by thermal analysis, which indicated a higher stability for the hydrogels compared to pure CMC. Rheological studies showed a gel-like behavior for all tested samples. Moreover, the obtained hydrogels presented a highly porous network, with fine and interconnected pores having the ability to absorb large amounts of water. Procaine was incorporated into hydrogels, and its release properties from the matrix were shown to depend on the polymer/crosslinker mixture ratio. Antimicrobial activity tests showed that the materials have antibacterial activity for both *E. coli* (Gram-negative) and *S. aureus* (Gram-positive) bacteria. Finally, the hydrogels were proved to be cytocompatible, considering the in vitro cell viability responses of over 96% towards fibroblast cells, revealing that the prepared hydrogels could be appropriate options for skin tissue engineering.

## 4. Materials and Methods

### 4.1. Materials

All chemicals obtained from commercial suppliers were used without further purification. Carboxymethyl cellulose (CMC), phytic acid (PA, 50 wt.% in water) and procaine hydrochloride (P) were supplied by Sigma Aldrich. In vitro cytocompatibility tests were performed on normal dermal fibroblasts (NHDF, PromoCell, Heidelberg, Germany). The cells were grown in alpha-MEM medium (Lonza, Basel, Switzerland) containing 10% fetal bovine serum (FBS, Gibco, Thermo Fisher Scientific, Waltham, MA, USA) and 1% penicillin–streptomycin–amphotericin B (10 K/10 K/25 μg, Lonza, Basel, Switzerland). An Ultra Clear TWF UV system was used to produce the deionized water that was used for the experiments.

### 4.2. Sample Preparation

#### 4.2.1. Preparation of CMC/PA Hydrogels

In this study, a series of three hydrogel variants was prepared by varying the feeding of PA into CMC (Table 5). In brief, CMC and deionized water were mixed and mechanically stirred for 24 h to prepare a 2.5% solution. Then, specific amounts of the crosslinking agent (PA, 50% by weight in water) were added dropwise under magnetic stirring to the CMC solution (5 mL). The reaction mixture became increasingly viscous shortly after the addition of PA as a crosslinking agent, and the morphology of the mixture gradually changed from solution to gel approximately 24 h after the start of the reaction. The prepared gel samples were further lyophilized to be characterized and loaded with the therapeutic agent.

#### 4.2.2. Preparation of Procaine-Loaded CMC/PA Hydrogels

Previously prepared and lyophilized gels were soaked in an aqueous solution of procaine (0.75% by weight) at a gel/drug mass ratio of 3/1, as presented in Table 5. The systems were maintained for 24 h under very slow stirring at room temperature (25 °C). In the case of CMC_PA = 10:1, another 3 mass ratios between the hydrogel and drug were taken into consideration: 4:1 (CMC_PA = 10:1_P_2); 5:1 (CMC_PA = 10:1_P_3); and 6:1 (CMC_PA = 10:1_P_4) (Table 5). The procedure allows the entire drug solution to be absorbed by the gel network. The bioactive samples were dried and refrigerated in dark containers until characterization.

### 4.3. Characterization 

#### 4.3.1. FTIR Spectroscopy

The chemical structure of the hydrogels was determined by means of FTIR spectroscopy. The samples, grounded onto KBr disks, were analyzed by using a Bruker spectrometer in vertex mode, with the absorption ranging from 400 cm^−1^ to 4000 cm^−1^, and the spectra were obtained at a resolution of 4 cm^−1^ as an average of 64 scans.

#### 4.3.2. Swelling Tests 

The prepared gels, in the lyophilized state, were tested for their swelling capacity by immersing them in a known amount of phosphate buffer solution (PBS) at pH = 7.4 until equilibrium was reached. Subsequently, the change in gel weight was measured at predetermined time intervals. During the weighing of the samples, PBS was removed from the surface with a filter paper so that only the weight of PBS incorporated in the hydrogel was taken into account. The equilibrium swelling degree (SDE) of the sample was calculated as follows:

SDE (%) = [(W_t_ − W_d)_/W_d_] · 100 (1)

where W_d_ and W_t_ are the weights of the dry and wet samples, respectively, at time t. Each experiment was performed in triplicate, and the average was obtained. 

#### 4.3.3. Rheological Studies

The rheological measurements were carried out at 25 °C with an MCR 302 Anton-Paar rheometer (Graz, Austria) using a plane–plane geometry (diameter of the upper plate of 50 mm, gap of 500 μm).

The oscillatory shear measurements were performed as a function of the oscillation frequency (ω), from 0.1 rad/s to 100 rad/s, for a constant strain of 1%, in the linear range of viscoelasticity that was established for each sample in amplitude sweep tests. The elastic (G′) and viscous (G″) moduli were determined as a measure of the stored energy and dissipated energy during one cycle of deformation, respectively. The loss tangent (tanδ = G″/G′) provides information on the degree of viscoelasticity of each sample. The shear viscosity (η) was determined in stationary shear flow conditions for shear rate values ($\dot{\gamma }$) in the range 0.01 s^−1^ to 100 s^−1^. 

#### 4.3.4. Scanning Electron Microscopy (SEM) Analysis

Freeze-dried samples fixed in advance by means of colloidal copper supports underwent SEM investigations. A thin layer of gold was spray-coated onto the materials (K Emitech550X). Next, a scanning electron microscope type Quanta 200, which operates at 30 kV with secondary electrons in high-vacuum mode, was used to analyze the covered area.

#### 4.3.5. Thermal Analysis

The thermal behavior of CMC/PA hydrogels was determined on a Netzsch STA 449 F1 Jupiter Simultaneous Thermal Analyzer. This system uses a nitrogen atmosphere and has a heating rate of 10 °C⋅min^−1^. Prior to that, the materials were kept in a controlled humidity atmosphere with the inorganic salt CaCl_2_. Accurate sample amounts (7.5–8 mg samples) were weighed and heated in an open Al_2_O_3_ crucible under a 50 mL/min^−1^ nitrogen flow rate. Runs were carried out in dynamic mode with a heating rate of 10 °C/min from room temperature up to 600 °C. Proteus^®^ software was used to collect the data. 

#### 4.3.6. In Vitro Studies of Procaine Release

In vitro release studies of the therapeutic agent were carried out in a 708-DS Dissolution Apparatus coupled with a Cary 60 UV-VIS spectrophotometer (Agilent Technologies, Santa Clara, CA, USA) by keeping the procaine-loaded hydrogels (~0.03–0.04 g) in 200 mL of phosphate buffer at pH 7.4 and 37 °C at a rotation speed of 100 rpm. Dissolution tests were performed for 24 h. Aliquots of the medium were withdrawn at predetermined time intervals and were analyzed at λmax = 292 nm by using a UV/VIS spectrophotometer. The polymeric matrix did not interfere with the spectrophotometric reading of the drug. The drug concentration was calculated based on the calibration curves determined at the same wavelengths.

The drug release data up to 60% of total drug release were fitted into the Korsmeyer–Peppas equation to determine the release mechanism (Equation (2)) [38].

M_t_/M_∞_ = k·t^n^ (2)

where M_t_/M_∞_ represents the fraction of the drug released at time t, while M_t_ and M_∞_ are the absolute cumulative amount of drug released at time t and the maximum amount released in the experimental conditions used, respectively, at the plateau of the release curves, k is a constant incorporating the characteristics of the macromolecular drug-loaded system, and n is the release exponent, which is indicative of the release mechanism.

#### 4.3.7. In Vitro Biocompatibility Assay

The biocompatibility of CMC/PA hydrogel samples was assessed using a CellTiter 96^®^ AQueous One Solution Cell Proliferation Assay (Promega, Madison, WI, USA), according to the manufacturer instructions and ISO 10993–5:2009(E). Normal human dermal fibroblasts (NHDF, PromoCell, Heidelberg, Germany) were seeded at a density of 0.5 × 10^5^ cells/mL into 96-well tissue culture-treated plates. After 24 h, the medium in each well was replaced with 100 μL hydrogel extracts (500 µg/mL, 250 µg/mL, 125 µg/mL, 62.5 µg/mL) or fresh complete medium (control). The extracts were performed in a complete cell culture medium at a concentration of 20 mg/mL for 96 h at 37 °C and then diluted 40× for cell incubation. Cells were incubated with extracts for 24 h, and 20 µL of MTS reagent was then added per well 3 h before reading the absorbance at 490 nm using a FLUOstar^®^ Omega microplate reader (BMG LABTECH, Ortenberg, Germany). Experiments were performed in triplicate, and treated cell viability was expressed as a percentage of the control cells’ viability. Graphical data were expressed as means ± standard error of the mean.

#### 4.3.8. Antimicrobial Activity

The antimicrobial activity screening of the samples was determined by disk diffusion assay [39,40] against two different reference strains: *Staphylococcus aureus* ATCC25923 (*S. aureus*) and *Escherichia coli* ATCC25922 (*E. coli*). All microorganisms were stored at −80 °C in 20% glycerol. The bacterial strains were refreshed on trypticase soy agar (TSA) at 37 °C, and the yeast strain was refreshed on Sabouraud dextrose agar (SDA) at 37 °C. Microbial suspensions were prepared with these cultures in sterile solution to obtain turbidity optically comparable to that of 0.5 McFarland standards. Volumes of 0.1 mL from each inoculum were spread onto TSA/SDA plates, and then the sterilized paper disks (6 mm) with an aliquot (50 μL) of the samples were added.

To evaluate the antimicrobial properties, the growth inhibition was measured under standard conditions after 24 h of incubation at 37 °C. All tests were carried out in triplicate to verify the results. After incubation, the samples were analyzed with SCAN1200^®^, version 8.6.10.0 (Interscience) and were expressed as the mean ± standard deviation (SD), performed with XLSTAT Ecology version 2019.4.1 software [41].

## Figures and Tables

**Table 1:** FTIR characteristic bands and their assignment for the studied hydrogels.

Wavenumber, cm^−1^			Type of Vibration	
CMC_PA = 20:1	CMC_PA = 10:1	CMC_PA = 6:1		
3457	3447	3435	–OH	CMC- and PA-related stretching modes
2932	2927	2927	C–H	
1742	1738	1734	COOH	CMC-related stretching modes
1645	1643	1641	P=O	PA-related stretching modes
1126	1130	1130	P–O–C	
1061–1007	1058–1012	1058–1012	C–O and C-O-C	CMC-related stretching modes

**Table 2:** Thermal parameters of CMC and CMC/PA hydrogels.

Sample	Heating Rate °C/min	Degradation Stage	T_onset_ °C	T_peak_ °C	W %	T_20_ °C	T_25_ °C
CMC	10	I II III IV residue	35 229 356 536	78 285 408 569	12.55 29.96 10.23 8.90 38.36	263	274
CMC_PA = 20:1	10	I II III IV residue	31 147 254 305	77 200 275 -	2.39 15.93 11.00 13.04 57.64	264	281
CMC_PA = 10:1	10	I II III IV residue	40 168 232 304	74 198 249 332	4.55 8.03 9.17 18.77 59.48	269	321
CMC_PA = 6:1	10	I II III IV residue	45 156 215 303	75 180 238 334	3.52 4.48 10.79 23.07 58.14	301	341

T_onset_—the temperature at which the thermal degradation starts; T_peak_—the temperature at which the degradation rate is maximum; T_20_, T_25_—the temperatures corresponding to 20% and 25% mass losses; W—mass losses.

**Table 3:** Kinetic parameters of procaine release in PBS buffer medium of pH 7.4 at a constant temperature of 37 °C.

Sample Name	n	R^2^_n_	k [min ^−n^]	R^2^_k_
CMC_PA = 20:1_1	0.229	0.9689	0.553	0.9976
CMC_PA = 10:1_1	0.338	0.9917	0.347	0.9979
CMC_PA = 10:1_2	0.344	0.9923	0.313	0.9932
CMC_PA = 10:1_3	0.418	0.9814	0.278	0.9979
CMC_PA = 10:1_4	0.381	0.9891	0.321	0.9965
CMC_PA = 6:1_1	0.568	0.9844	0.188	0.9940

n—release exponent; k—release rate constant; R^2^_n_ and R^2^_k_—correlation coefficients corresponding to the slope obtained for determination of n and k.

**Table 4:** Antimicrobial activity of the tested CMC/PA hydrogels against reference strains (mm).

	*S. aureus*		*E. coli*	
	Inhibition Zone (mm)		Inhibition Zone (mm)	
CMC_PA = 20:1	8.80 ± 0.28	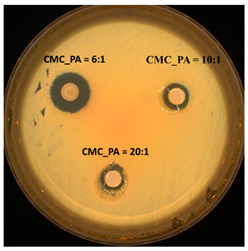	9.10 ± 1.69	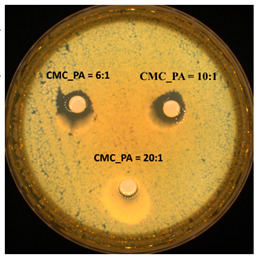
CMC_PA = 10:1	10.60 ± 0.56		10.15 ± 1.06	
CMC_PA = 6:1	12.00 ± 0.42		17.50 ± 0.14	

**Table 5:** Chemical composition of the CMC hydrogels in relation with the PA crosslinker content.

**Sample Code**	**CMC, mL**	**PA, µL**
CMC_PA = 20:1	5	250
CMC_PA = 10:1	5	500
CMC_PA = 6:1	5	750
**Procaine-Loaded Samples**		
**Sample Code**	**Hydrogel Sample, g**	**Procaine, g**
CMC_PA = 20:1_P_1	0.036	0.0120
CMC_PA = 10:1_P_1	0.036	0.0120
CMC_PA = 10:1_P_2	0.036	0.0090
CMC_PA = 10:1_P_3	0.036	0.0075
CMC_PA = 10:1_P_4	0.036	0.0060
CMC_PA = 6:1_P_1	0.036	0.0120

## Data Availability

Not applicable.
